# Does Embryo Culture Medium Influence the Health and Development of Children Born after *In Vitro* Fertilization?

**DOI:** 10.1371/journal.pone.0150857

**Published:** 2016-03-23

**Authors:** Céline Bouillon, Roger Léandri, Laurent Desch, Alexandra Ernst, Céline Bruno, Charline Cerf, Alexandra Chiron, Céline Souchay, Antoine Burguet, Clément Jimenez, Paul Sagot, Patricia Fauque

**Affiliations:** 1 Service de Médecine et Biologie de la Reproduction, Centre Hospitalier Régional Universitaire de Tours, Tours, France; 2 Centre d’Assistance Médicale à la Procréation, Hôpital Paule de Viguier, Groupe de Recherche en Fertilité Humaine, EA 3694, Toulouse, France; 3 Laboratoire de Biologie de la Reproduction, Hôpital de Dijon, Equipe GAD, Génétique des Anomalies du Développement, EA 4271, Université de Bourgogne, Dijon, France; 4 LEAD—CNRS UMR 5022, Université de Bourgogne, Pôle AAFE, Dijon, France; 5 Laboratoire de Biologie de la Reproduction, SELAFA BIOFFICE—Clinique Jean Villar, Bruges, France; 6 Service de Pédiatrie, Hôpital de Dijon, Université de Bourgogne, Dijon, France; 7 Service de Biologie de la Reproduction-CECOS, Institut des maladies neurodégénérativesCNRS UMR 5293, Université de Bordeaux, Bordeaux, France; 8 Service de Gynécologie-Obstétrique, Médecine Fœtale et Stérilité Conjugale, Hôpital de Dijon, Université de Bourgogne, Dijon, France; Friedrich-Loeffler-Institute, GERMANY

## Abstract

In animal studies, extensive data revealed the influence of culture medium on embryonic development, foetal growth and the behaviour of offspring. However, this impact has never been investigated in humans. For the first time, we investigated in depth the effects of embryo culture media on health, growth and development of infants conceived by *In Vitro* Fertilization until the age of 5 years old. This single-centre cohort study was based on an earlier randomized study. During six months, *in vitro* fertilization attempts (No. 371) were randomized according to two media (Single Step Medium—SSM group) or Global medium (Global group). This randomized study was stopped prematurely as significantly lower pregnancy and implantation rates were observed in the SSM group. Singletons (No. 73) conceived in the randomized study were included (42 for Global and 31 for SSM). The medical data for gestational, neonatal and early childhood periods were extracted from medical records and parental interviews (256 variables recorded). The developmental profiles of the children in eight domains (social, self-help, gross motor, fine motor, expressive language, language comprehension, letter knowledge and number knowledge – 270 items) were compared in relation to the culture medium. The delivery rate was significantly lower in the SSM group than in the Global group (*p*<0.05). The culture medium had no significant effect on birthweight, risk of malformation (minor and major), growth and the frequency of medical concerns. However, the children of the Global group were less likely than those of the SSM group to show developmental problems *(p* = 0.002), irrespective of the different domains. In conclusion, our findings showed that the embryo culture medium may have an impact on further development.

## Introduction

Experimental animal studies on maternal diet during early embryonic development clearly showed that even subtle changes in the nutritional environment could alter phenotypes in offspring (like insulin resistance and vascular system dysfunction with high blood pressure) [1]. Similarly, it has been shown that individuals conceived during a period of famine have an increased risk of diseases like obesity, coronary artery disease and cognitive schizophrenia [2–4]. In addition, there is a lot of evidence from mouse studies that environmental culture conditions could affect the birthweight of offspring and have later consequences. Some authors recently reported that the type of culture medium used in human reproduction may also influence the birthweight of the children thus conceived [5–8]. Thus, the health of children born following Assisted Reproductive Technologies (ART) needs to be monitored with regard to the efficacy of the embryo culture medium used.

In our lab, an earlier randomized study was conducted to assess the effectiveness of two different culture media on *In Vitro* Fertilization (IVF) outcomes. However, six months later, this study was prematurely stopped as one medium was found to be significantly underperforming (in terms of pregnancy rate).

Thus, the purpose of the current study was to investigate in depth the effects of this underperforming embryo culture medium on children’s health at birth and in the longer term, until the age of 5 years old. Our findings showed that embryo culture may have an impact on further development. These results confirm that the preimplantation period is a critical window of development. This is aligned with the consensus hypothesis that ART procedures increase epigenetic perturbations, potentially causing long-term health outcomes.

## Materials and Methods

### Study design and Population

This single-centre cohort study is based on an earlier randomized study conducted in 2008 at the University Hospital of Dijon, which compared the IVF outcomes following use of the two media: Global medium (LifeGlobal) and Single Step Medium (SSM, Irvine Scientific), the components are presented in Table 1. Indeed, during this study, both embryo culture media were randomly used from the day of oocyte retrieval to day 2 or day 3; all other culture conditions in the laboratory were identical. Patients were randomized by the embryologist on the day the cycle was started according to a computer-generated randomization table. Clinicians performing the embryo transfer and patients were blinded for the allocation. However, as this randomized study showed that Global medium was significantly superior to SSM medium in terms of pregnancy and implantation rates, the study was stopped 6 months later.

**Table 1:** Components of both culture media according to published analyses*.

Component Type			*Global medium*	*SSM medium*
**Salts and Ions**		Sodium Chloride	+	+
		Potassium Chloride	+	+
		Magnesium Sulfate	+	+
		Calcium Chloride	+	+
		Potassium Phosphate	+	+
**Buffer**		Sodium Bicarbonate	+	+
**Energy Substrates**		Glucose	+	+
		Sodium Pyruvate	+	+
		Sodium Lactate	+	+
**Amino Acids**	***Essential amino acids***	Arginine	+	+
		Cysteine	+	+
		Histidine	+	+
		Isoleucine	+	+
		Leucine	+	+
		Lysine	+	+
		Methionine	+	+
		Phenylalanine	+	+
		Threonine	+	+
		Tryptophan	+	+
		Tyrosine	+	+
		Valine	+	+
	***Non-essential amino acids ***	Alanine	+	+
		Asparagine	+	+
		Aspartic acid	+	+
		Glutamic acid	+	+
		Glycine	+	+
		Proline	+	+
		Serine	+	+
		Taurine	-	+
	***Di-peptide***		Glycyl-L-Glutamine	Alanyl-L-Glutamine
**Chelator**		EDTA	+	+
**Indicator**		Phenol Red	+	+
**Antibiotic**		Gentamicin	+	+

* Zhao et al., 2013; Morbeck et al., 2014

Global medium was used for 179 attempts and SSM medium for 192 attempts of conventional IVF or ICSI (IntraCytoplasmic Sperm Injection). The difference corresponded to cases without embryo transfer (ovarian hyperstimulation syndrome, high progesterone hormone levels and no oocyte at retrieval). For the present study, all singleton live births after embryo transfers of this randomized study were included. Altogether, 73 singleton births occurred including 42 for Global medium and 31 for SSM medium.

All participating couples had given their written informed consent to participate in the earlier randomized study and also in the children health study. This consent procedure was approved by the Institutional Review Board of Burgundy in France, and registered under number “2008-A01491-54”.

### IVF procedure and outcome measurements

The choice between ICSI and IVF as the fertilization method depended upon semen sample characteristics and the history of the couples involved.

Oocytes and embryos were individually cultured in 30μl of either Global or SSM, both of which were supplemented identically with Human Serum Albumin 10%, Irvine Scientific) at 37°C under a humidified 3-gas atmosphere (5% CO_2_, 5% O_2_ and 90% N_2_). The mineral oil used to recover the culture droplets (Nidacon), culture dishes (BD Falcon) and incubators were also identical for both groups. The pH of the equilibrated media was around 7.3.

Depending on the age of the women, the number of previous cycles, and the number and quality of embryos available, one or two embryos were transferred at day 2 or day 3.

### Data collection

The collected items were established in collaboration with the Officials of ELFE study (French longitudinal study of children) [9]. Deliveries occurred in 11 maternity hospitals. Gestational and neonatal medical data were extracted from the medical record on site (97 variables recorded). Of the 73 deliveries, there was only one delivery for which we were unable to obtain information.

Regarding the data on the long-term health, growth and development of children, couples were first contacted by mail. In the absence of a refusal to continue to participate, they were contacted for a telephone interview. Of the 73 couples, seven could not be reached. All of the couples contacted agreed to the interview, and their responses were based on the national medical health record book (*carnet de santé*) of the children (159 variables recorded). This child’s *carnet de santé* includes compulsory medical examinations performed at 8 days, at 2, 4, 9, and 24 months, and then at 3 and 4 years. Weight, height, and head circumference were recorded at the age of 2 months, 4 months, 9 months, 24 months, 3 and 4 years. Head circumference was only measured until the age of 24 months. Post-natal weight and height measurements were expressed as standard deviation scores, normalized for age and gender (according to AUDIPOG database [10]). Any hospitalization, surgery or disease was noted. The various malformations detected at birth and after the neonatal period were classified as minor or major malformations according to the EUROCAT (European Surveillance of Congenital Anomalies) classification [11].

Around five years after delivery, parents completed the French version of the Child Development Inventory (CDI) based on 270 items [12], which aims to assess the developmental profile of children in eight domains, including social, self-help, gross motor, fine motor, expressive language, language comprehension, letter knowledge and number knowledge. By the CDI analysis, two measurements of the children’s development were provided: the developmental age for each domain, which corresponds to the age at which behaviors first appear in children in general and the general developmental score, which takes into account the chronological age of children and includes 70 items extracted from all 270 items previously demonstrated to be age-discriminating (Instructions and examples of items are provided in S1 File). This questionnaire has been selected based on its psychometric properties showing a high sensitivity and specificity as well as a good predictive value [12–14]. The CDI has been identified as a useful and cost-effective screening measure for determining developmental outcomes among high-risk infants [14].

### Statistical Analysis

Before comparing the effects of both culture media on the health of children, the homogeneity between the two groups (Global and SSM media) was tested for maternal age, distribution between IVF and ICSI, indication and attempt number. A Chi^2^ test was used for binary variables. The Fisher test was used to compare small sample sizes. For continuous variables, analysis of variance was performed. If necessary, a Kruskal-Wallis test was applied. Since the CDI results were not normally distributed, data were first transformed to normality, for both the developmental age and the general developmental score. The absence of any between-group difference for the parental socio-economic level (according to the INSEE database, i.e. the French National Institute of Statistics and Economic Studies) was first verified using Chi^2^ test. Correlations between the CDI domains and gender, term of birth, mother and father’s socioeconomic status were explored. In case of significant correlation, these variables were included as covariable. Regarding the developmental profile in the eight domains of the CDI, the effect of the culture medium was explored in a blinded manner using repeated measures ANOVA. Similar analyses were conducted to investigate the effect of the fertilization method. Concerning the general developmental score, a t-test for independent samples was used. The difference was considered significant if *p*≤0.05.

## Results

### Preimplantation and prenatal periods

In the randomized study conducted in 2008, the implantation and delivery rates were significantly lower in the SSM group than in the Global group (*p*<0.05—Table 2). For each group, the characteristics of the parents and cycles of the 73 singletons born after embryo transfer are shown in S1 Table. For these singletons, no difference was found between the groups except for early embryo development (embryos in the SSM group exhibited significantly more fragmentation and, their development was significantly late in comparison with embryos cultured in Global medium, data not shown).

**Table 2:** Pregnancy outcomes.

	Global group	SSM group	Rate Ratio	p
	*(No*. *179)*	*(No*. *192)*	*(95% CI)*	
Implantation (% per embryo transferred^a^)	82 (29.1%)	48 (16.3%)	1.78 [1.31–2.43]	0.001
Clinical pregnancies (% per cycle^b^)	66 (36.9%)	42 (21.9%)	1.68 [1.22–2.33]	0.001
Twin pregnancies (% per clinical pregnancy)	8 (12.1%)	4 (9.5%)	1.27 [0.41–3.96]	0.76
Early spontaneous abortion (% by positive pregnancy test)	18 (24.7%)	9 (18.0%)	1.37 [0.68–2.78]	0.38
Ectopic pregnancy (% by positive pregnancy test)	1 (1.4%)	0 (0%)	NC	-
Therapeutic abortions (% by positive pregnancy test)	1* (1.4%)	0 (0%)	NC	-
Delivery of live-born children (% per cycle^c^)	50 (27.9%)	35 (18.2%)	1.53 [1.05–2.23]	0.03
Delivery of singleton (% per total number of live birth deliveries)	42 (84.0%)	31 (88.6%)	0.95 [0.80–1.13]	0.75
Delivery of twin (% per total number of live birth deliveries)	8 (16.0%)	4 (11.4%)	1.40 [0.46–4.26]	0.75

Data are presented as numbers (%)

*No*.: number of cycles

NC: Not calculable

*: for exencephaly

^a^: The implantation rate was the ratio between the number of gestational sacs and the number of transferred embryos.

^b^: Clinical pregnancy was determined by the presence of an intrauterine gestational sac with a foetal heartbeat on ultrasound examination 4–5 weeks after the embryo transfer.

^c^: The delivery rate was the ratio between the number of deliveries and the number of embryo transfers.

For the gestational complications and distribution of the delivery mode, no significant difference was found between the groups (S2 Table).

### Birthweight and malformations

At birth, no difference was noted between the two groups for gestational age, sex distribution, proportion of children admitted to a neonatal intensive care unit and days of hospitalization (S3 Table). The birthweight of singletons was 3131g ± 505g and 3145g ± 448g for the Global and SSM groups, respectively. Taking into account the criteria considered relevant following univariate analyses (S1 Table), namely male smoker, gestational diabetes, term birth and gender, the culture medium had no significant effect on birthweight (*F*(1, 68) = 1.16, *p* = 0.28; η^2^_P_<1%). None of the offspring had a very low birthweight (<1500g) or high birthweight (>4500g).

Defects detected in singletons were classified as minor and major malformations according to the EUROCAT classification (Table 3, S2 File). Statistical analysis showed no difference between the groups regarding defects, whether minor or major.

**Table 3:** Malformations.

		Global group*	SSM group*
		*(No*. *40)*	*(No*. *31)*
**Major malformations (according to EUROCAT)**	Detected during the neonatal period	2 (5.1%)	1 (3.2%)
	Detected after the neonatal period	1 (2.8%)	1 (3.3%)
**Minor malformations**	Detected during the neonatal period	7 (18.0%)	3 (9.7%)
	Detected after the neonatal period	9 (25.0%)	7 (23.3%)
**Major malformations by organ type**	Cardiac^a^	2 (5.6%)	1 (3.3%)
	Nervous system^b^	0 (0%)	1 (3.3%)
	Limbs^c^	1 (2.8%)	0 (0%)

Available data are presented as numbers (%).

*No*.: number of singletons

^a^All cardiac malformations were ventricular septal defect

^b^The nervous system abnormality was a macrocrania with hydrocephalus but without intracranial hypertension and with a normal neurological development of the child.

^c^ The limb abnormality was a bilateral syndactyly of the first knuckle of the 2nd and 3rd toes.

* Statistical analysis showed no difference between the groups, p>0.05

### Growth and medical concerns

No significant differences between the groups emerged for weight, height, body mass index and head circumference measured at the age of 9 months, 24 months and 4 years (S1 Fig).

Up to 4 years old, various medical histories were studied regarding hospitalization, medical or surgical treatment and different chronic diseases (Table 4, S3 File). No differences between the groups were found.

**Table 4:** Singletons' medical history at 4 years old.

		Global group*	SSM group*
		*(No*. *36)*	*(No*. *30)*
**Age (years)**		4.2 (0.2)	4.1 (0.2)
**Weight (kg)**		16.2 (2.3)	16.0 (1.9)
**Height (cm)**		103.4 (4.3)	104.2 (4.1)
**BMI**		15.1 (1.3)	14.8 (1.1)
**Asthmatic medication**		5 (13.9%)	5 (16.7%)
**Treatment> 1 month**		18 (50.0%)	14 (46.7%)
**Eczema > 1 flare**		7 (19.4%)	9 (30.0%)
**Psychotherapy**		6 (16.7%)	4 (13.3%)
**Hospitalizations (night)**		10 (27.8%)	13 (43.3%)
**Hospitalizations (day)**		9 (25.0%)	5 (16.7%)
**Chronic diseases**		8 (22.2%)	6 (20.0%)
	Cardiac	2 (5.6%)	1 (3.3%)
	Pulmonary (asthma)	4 (11.1%)	4 (13.3%)
	Neurological	1 (2.8%)	1 (3.3%)
	ENT with secondary deafness	1 (2.8%)	1 (3.3%)
	Digestive	1 (2.8%)	0 (0%)
	Haematological	1 (2.8%)	0 (0%)
	Other diseases	0 (0%)	1 (3.3%)
**Surgery**		8 (22.2%)	5 (16.7%)
	Circumcision	1 (2.8%)	3 (10.0%)
	Ventilating tube treatment	4 (11.1%)	3 (10.0%)
	Tonsillectomy	1 (2.8%)	1 (3.3%)
	Adenoid removal	2 (5.6%)	1 (3.3%)
	Other surgeries	3 (8.3%)	0 (0%)

Available data are presented as numbers (%) or mean (SD)

BMI: Body Mass Index, ENT: Ear Nose and Throat, No.: number of singletons

* Statistical analysis showed no difference between the groups, p>0.05

### Development

No significant difference was found between the groups regarding the socioeconomic status of mothers (*p* = 0.45) or fathers (*p* = 0.68).

A first set of analyses was conducted on the developmental age of 55 children (31 from the Global group and 24 from the SSM group, established on complete data) in each CDI domain. Statistical analysis revealed a main effect of the culture medium (*F*(1, 53) = 5.32, *p* = 0.02, η^2^_P_ = 0.09), with a significantly higher developmental age in children from the Global group. In other words, children from the Global group overall obtained better developmental scores than those from the SSM group, in developmental domains including social skills, self-help, gross and fine motor skills, expressive language, language comprehension, as well as letter and number knowledge.

To control for the influence of potential confounding factors, correlations between gender, term birth, mother and father’s socioeconomic status with the CDI domains were explored (S4 Table). Based on these results, mother’s socioeconomic status was included as a covariable in ANOVA analysis. The effect of culture medium type on developmental age was still significant (F(1, 52) = 4.06, *p* = 0.04, η^2^_P_ = 0.07), showing a higher developmental age in children from the Global Group than in those from the SSM group. No significant culture medium x CDI domains interaction was obtained (*F*(7, 371) = 1.98, *p* = 0.06, η^2^_P_ = 0.03). The absence of any significant interaction means that the effect of culture medium on developmental problems is the same in every CDI domain.

**Fig 1:**
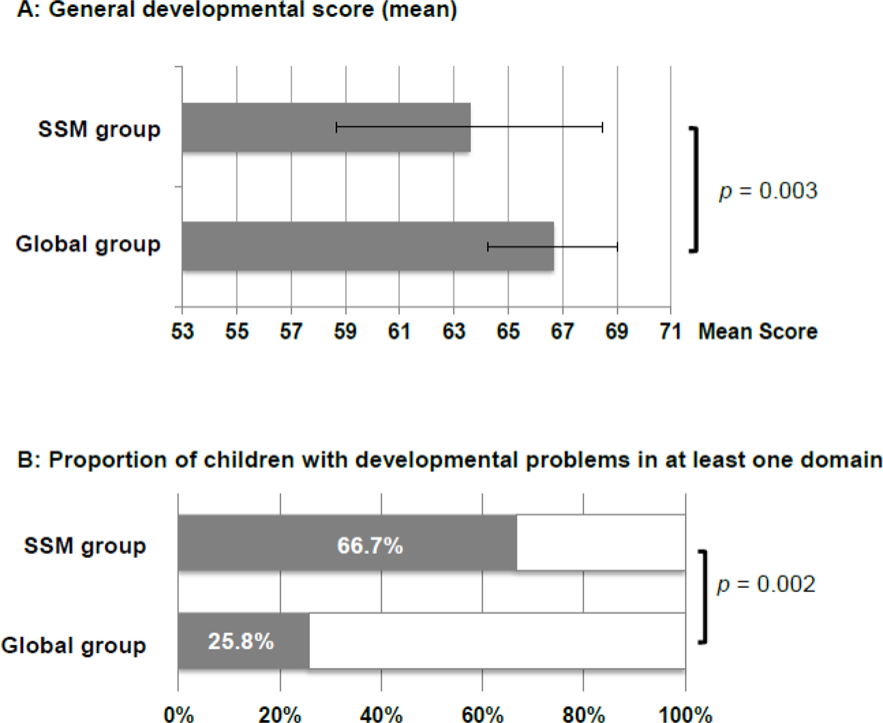
Development. General developmental score (A) and proportion of children with developmental problems in at least one out of eight domains according to the CDI norms (B) in Global and SSM groups.

The second set of statistical analyses focused on the general developmental score, which provides a general developmental indicator taking into account the chronological age, with no distinction of particular developmental domains (see S1 File for more details on the scoring). The comparison of this measure between the groups showed a significant difference (*t* = 3.04, *p* = 0.003), with a higher score for the Global group than for the SSM group (Fig 1).

A complementary analysis was run to explore the number of children with significant developmental problems (according to the CDI norms) in each group. The number of children presenting with developmental problems in at least one CDI domain was significantly lower in the Global group (8 out of 31 children) than in the SSM group (16 out of 24 children) (*Chi*^*2*^ = 9.18, *p* = 0.002—Fig 1).

The comparison of the fertilization method yielded no significant results (for each CDI domain: *F*(1, 53) = 0.71, *p* = 0.40, η^2^_P_ = 0.01 or regarding the general developmental score: *p* = 0.55) and no significant fertilization method x CDI domains interaction was found (*F*(7, 371) = 1.23, *p* = 0.28, η^2^_P_ = 0.02).

## Discussion

To our knowledge, no prior studies have explored the longer-term effects of culture media on health, growth and development of infants until the age of 5 years old. Our data highlight significant differences on the developmental age. These findings of the effect of embryo culture medium are crucial and raise important points.

Studies published so far have mainly focused on the health of children according to the IVF method used [15]. The influence of the culture medium was assessed with regard to gestational development, birthweight and health at birth [5]. Only one recent study on culture media has been published, and this concerned weight during the first two years of life [16]. However, in animal studies, extensive data are available and reveal the influence of the type of culture medium on embryonic development and foetal growth of offspring [5,17,18]. In our study, we observed poorer embryo scoring with SSM than with Global. It is important to know what differences in culture media composition may explain this negative impact on embryo development. We can exclude the protein source, as the supplements were the same in both media. From manufacturers’ data and previous published analyses, the composition of both media used in this study seems to be roughly identical except for two components [19,20]. First, SSM medium contains the amino acid taurine whereas Global medium does not. Taurine has been added to culture media for its antioxidant properties even though its ability to increase blastocyst formation in humans has not been proven [21]. A recent study performed in chick embryos even suggested that exogeneous taurine increased brain oxidative stress and apoptosis during embryo development [22]. Second, glutamine, a deliberately omitted labile amino acid, is supplied in both media by different heat-stable di-peptides (Alanyl-L-Glutamine (AlaGln) in SSM and Glycyl-L-Glutamine (GlyGln) in Global) in order to prevent ammonium production, which has long been known to retard foetal development and cause neural tube defect exencephaly [23] even after short exposure especially during the cleavage stages [24]. However, in line with our results, the lower efficacy of AlaGln as compared with GlyGln has also been reported in mouse preimplantation embryos [25]. Potentially, ammonium release could be different depending on the di-peptide form used. Recently, Wale and Gardner showed that embryo culture performed under 5% oxygen induced glutamine production and could alleviate ammonium stress in the mouse embryo [26]. In the current study, 5% oxygen was employed. Another important method to reduce ammonium production is to titrate down the levels of amino acids in the medium [27]. Regrettably, as the manufacturers didn’t disclose the specific concentration of components, a valid analysis of the levels of amino acids in the two media is currently impossible.

In humans, studies were essentially based on early embryo development or implantation rates [28], and those analysing birthweight and post-natal growth remain limited [5,16]. However, some studies, again revealed that the composition of the culture medium could affect early embryonic and foetal development and even birthweight [7,8]. Meta-analyses reported, after correction for confounding factors, that ART-conceived singletons also had a higher risk of prematurity and perinatal morbi-mortality than children conceived naturally [29,30]. In our study, after identical adjustments, no difference was highlighted between the two culture media at this step. Finally, we found no increased risk of either minor and major malformations or physical health problems until the age of five years in singletons from the SSM group although this medium was underachieving. We have to keep in mind that this absence of difference is probably due to the fact that the size of our children cohort is relatively small to evidence non-major differences between groups. However, this study revealed that the children from the Global group were significantly less likely to display developmental problems than were those of the SSM group. Importantly, this effect of culture medium on child development was the same in every CDI domain, including social skills, self-help, gross and fine motor skills, language comprehension, expressive language, letter and number knowledge. The method used in this work to determine the developmental outcomes has been selected based on its psychometric properties showing a high sensitivity and specificity as well as a good predictive value (i.e. in comparison with standard measure of developmental status such as neuropsychological tests) [12–14]. To our knowledge, no study evaluated the developmental and cognitive status of ART-singletons according to the type of culture medium. However, some studies on mental and motor development of ART-children showed that children were more likely to have delayed mental and motor development and a lower intelligence quotient [31–33] whereas other studies reported no adverse cognitive and motor outcomes [32,34–36].

The current study provides a valid and detailed comparison. Indeed, the initial randomized study performed in a single centre over a short period of time ensured that the environment of the lab, especially the culture equipment (i.e. incubator, plastic dishes, oil, etc.…) was similar. In addition, the participation was very high (90.4%) as was the completeness of data (89.6%). The absence of significant differences for health issues should not reassure us. Differences in the health status may occur later, which is why this cohort study requires a longer follow-up. Indeed, Ceelen and colleagues reported an increase in the sum of skinfolds and a higher risk of cardiovascular disease at age 8–18 years in IVF-children than in children from the general population (2009) [37]. In addition, several authors reported that new-borns that are either too small or too big may be vulnerable to heart disease, hypertension, type II diabetes and obesity in adulthood [38,39]. As reported in animal models, the embryo culture step itself could perturb embryo metabolism and gene expression [40] and have consequences on behaviour [41,42]. Even though the developmental delay at five years observed in SSM group may disappear later, it could be the first sign of more long-term neuromotor developmental concerns.

These embryonic responses to early environmental stress could be kept in memory and provoke adverse effects in infancy and even in adulthood, potentially through epigenetic changes (such as DNA methylation). Indeed, in recent years, it has been shown that the risk of epigenetic disorders is greater in ART-children than in naturally conceived children [43] and that any stress during embryo culture can cause epigenetic changes in humans and in animals [44–48] which are potentially responsible for the occurrence of disease not only at birth but also in adulthood [49]. Furthermore, in a recent study some epigenetic errors can still be observed during childhood in ART infants [50].

Thus, it would be interesting to carry out studies comparing the epigenetic landscape of children born following the use of both media. Regions involved in neuronal development should be specifically studied since DNA methylation was recently found to be altered in the sperm of fathers of infants with autism signs [51].

## Conclusions

For the first time, we have observed that embryo culture medium can have an impact on development in humans. These findings are crucial and further investigations are needed. Indeed, they could also enhance our knowledge about maternal nutrition during the periconception period. Media are optimized in animal models. However, in view of our results and the literature, the precise formulation of culture media should be disclosed and we recommend more evaluations dedicated to human embryo culture before their commercialization, for example by including epigenetic assays on mouse embryos. Our findings also plead for both neonatal (only demanded in France) and long-term follow-up of ART-children.

## Supporting Information

S1 Fig **Comparison of singletons’ weight (A), height (B), BMI (C) and head circumference (D) at the age of 9 months, 24 months and 4 years old.**
(TIF)

S1 File Instructions and examples of items for each domain of the CDI. (DOCX)

S2 File Details on malformations. (DOCX)

S3 File Details on growth and medical concerns. (DOCX)

S1 Table Parental and cycle characteristics of singletons. (DOCX)

S2 Table Maternal health during pregnancy. (DOCX)

S3 Table Neonatal data of singletons. (DOCX)

S4 Table Correlations between gender, term birth, mother and father socio-economic status with the CDI domains. (DOCX)
