# Supplementary figures and images for: Does Embryo Culture Medium Influence the Health and Development of Children Born after In Vitro Fertilization?
Source: PLoS One. 2016 Mar 23;11(3):e0150857. doi: 10.1371/journal.pone.0150857 (PMC4805279; doi:10.1371/journal.pone.0150857)

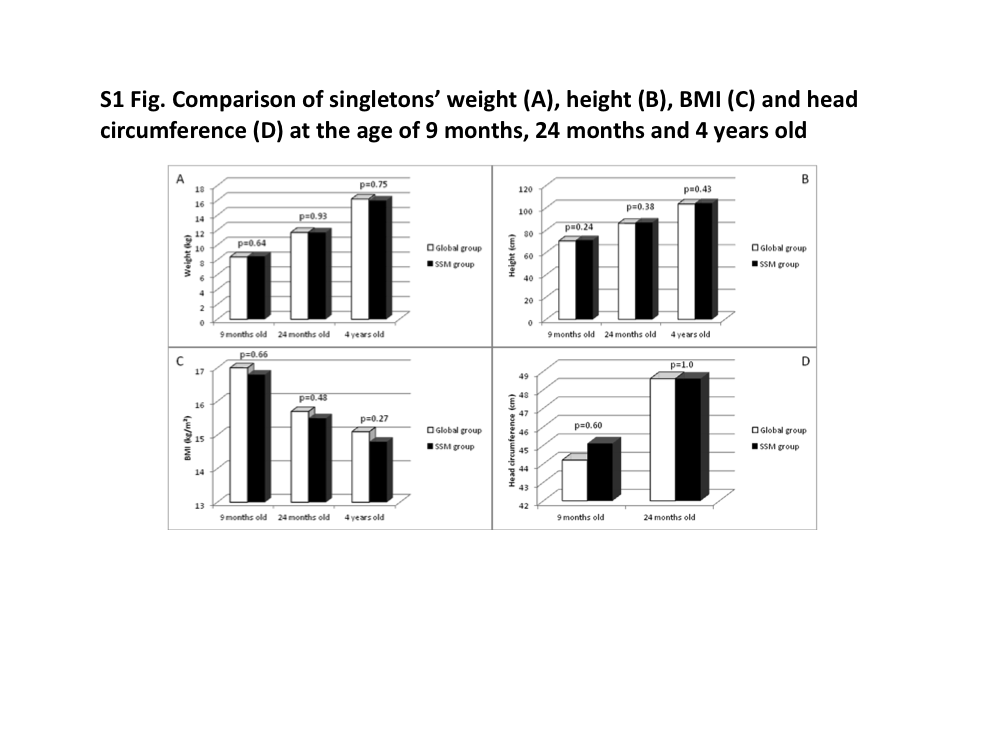

Supplement: S1 Fig — Comparison of singletons’ weight (A), height (B), BMI (C) and head circumference (D) at the age of 9 months, 24 months and 4 years old. (TIF) [file pone.0150857.s001.tif]
