# Supplementary material for: Does Embryo Culture Medium Influence the Health and Development of Children Born after In Vitro Fertilization?
Source: PLoS One. 2016 Mar 23;11(3):e0150857. doi: 10.1371/journal.pone.0150857 (PMC4805279; doi:10.1371/journal.pone.0150857)
Supplement: S1 File — (DOCX) [file pone.0150857.s002.docx]

**S1 File. Instructions and examples of items for each domain of the CDI**

**A Word to Parents**

Parents’ observations of their children can provide important information about their development. The Child Development Inventory includes statements that describe young children’s behavior. The Inventory asks you to report what your child is doing. It can help to understand your child’s development and needs.

**Instructions**

Please read each statement carefully. Use the Yes-No answer sheet to record your answers. If you need help reading or understanding the items or have any questions, please ask.

Fill in your CHILD’S NAME (print last name, skip one box, then first name), SEX, BIRTH DATE, and the DATE you COMPLETED this inventory.

Also, complete the FAMILY INFORMATION section, including your child’s SPECIAL PROBLEMS or DISABILITIES, if any.

Answer YES or NO to each statement in the booklet to report what you have seen your child doing.

Answer YES — if the statement describes your child’s present behavior. Also, answer YES if the behaviour is something that your child used to do, like crawling and babbling.

Answer NO — if the statement does not describe your child’s behavior. Also, answer NO if the behaviour is something that your child is only just beginning to do or only does sometimes.

Answer YES by filling in the circle marked Y on the answer sheet; answer NO by filling in the circle marked N.

Use a pencil so that you can erase an answer if you want to change it. Be sure the number of the statement in the booklet matches the number you are marking on the answer sheet.

If your child is younger than two years, many of the statements will not describe his or her behavior.

Even so, read all the statements and answer every statement with YES or NO.

***Examples of items for each domain of the CDI. The total number of items per domain is indicated in brackets.***

- **Social (40 items)**

*Examples:*

- Recognizes familiar adults and reaches for them
- Shows sympathy to other children, tries to help and comfort them
- Speaks positively about self — says, “I’m good,” “I’m big,” etc
- Sometimes will sacrifice his(her) own wishes for the benefit of the group
- Plays simple board games such as checkers
- **Self-help (40 items)**

*Examples:*

- Feeds self a cracker or cookie
- Tries to put on shoes. Or puts them on
- Washes self in bathtub — may need a little help
- Goes to the toilet without help; wipes self, flushes toilet, and washes hands
- Ties shoelaces
- **Gross motor (30 items)**

*Examples:*

- Pulls self to standing position. Or gets self to standing
- Kicks a ball
- Jumps from steps with feet together. Or used to
- Rides around on tricycle using pedals
- Does cartwheels
- **Fine motor (30 items)**

*Examples:*

- Uses two hands to pick up large objects
- Builds a tower of five or more blocks
- Scribbles with a circular motion. Or used to
- Cuts across paper with scissors from one side to the other
- Colors within the lines in a coloring book
- **Expressive language (50 items)**

*Examples:*

- Calls you “Mama” or “Dada” or similar name
- Names a few familiar objects in picture books
- Uses the word “not” in sentences
- Puts two sentences together with the words “and,” “or,” or “but.”
- Names the days of the week in correct order
- **Language comprehension (50 items)**

*Examples:*

- Usually comes when called
- Hands a toy to you when asked
- Identifies at least one color by name correctly
- Takes part in conversations, both talking and listening in turn.
- Knows right hand from left
- **Letter knowledge (15 items)**

*Examples:*

- Asks what signs say, such as road signs, advertising, etc.
- Recites the alphabet, in order, without help
- Prints a few simple words from a copy
- Prints two or more simple words from memory
- Attempts to read words by separating them into parts, for example, “el-e-phant.”
- **Number knowledge (15 items)**

*Examples:*

- Points to or names the “bigger” of two objects when asked
- Counts three or more objects
- Recognizes and names a few single numbers
- Recites numbers in order from 1 to 30
- Tells time: Reads clock in hours and minutes

**Scoring**

Two measures were considered: the developmental age for each CDI domain and the global developmental score.

The developmental age for each CDI domain corresponds to the age at which the behavior described first appears in a large enough proportion of children to be considered reasonably characteristic of that age. The CDI defines the developmental age of an item as the age at which at least 75 percent of parents answered YES to the statement.

The global developmental score (Duyme et al., 2011) is formed by selecting 10 items for each of the 6 developmental scales (60 items) and 5 items for each of the 2 learning scales (10 items; letter and number knowledge). These 70 items were retained as they were demonstrated to be age-discriminating. The total number of points obtained corresponds to a developmental age, according to the same procedure as that of the other scales. Then, the global developmental score is calculated based on the chronological age of children (months between birth and date of parents’ reply), with the following formula: ([developmental age/chronological age] ×100).

Duyme M, Zorman M, Tervo R, Capron C. French norms and validation of the Child Development Inventory (CDI): Inventaire du Developpement de l'Enfant (IDE). Clinical pediatrics 2011;50:636-47.
