# Supplementary material for: Does Embryo Culture Medium Influence the Health and Development of Children Born after In Vitro Fertilization?
Source: PLoS One. 2016 Mar 23;11(3):e0150857. doi: 10.1371/journal.pone.0150857 (PMC4805279; doi:10.1371/journal.pone.0150857)
Supplement: S2 File — (DOCX) [file pone.0150857.s003.docx]

S2 File. Details on malformations

At birth, seven and three minor defects were discovered in the Global and SSM groups, respectively. In the Global group, we identified one infant with bilateral testicular ectopia, one with a birthmark on the forehead and on the thigh, four with reducible varus feet and one with plagiocephaly. In the SSM group, one newborn had reducible varus feet, one had a reducible varus foot and retrognathia, and the third one had a very moderate and reducible umbilical hernia. Two major malformations were found at birth in the Global group: ventricular septal defect (VSD) (closed at the age of 6 months) and bilateral syndactyly at the first knuckle of the 2nd and 3rd toes, which posed no problem for the acquisition of walking later. In the SSM group, one major malformation was detected at birth: macrocrania with hydrocephalus but without intracranial hypertension. The child was followed until the age of 18 months. His neurological development was normal and without developmental delay.

Nine minor defects were detected in the Global group: two cases of flat feet and one case each of retractile testicles, phimosis, hip dysplasia that did not require treatment, varus feet, operated bilateral trigger thumbs, operated unilateral inguinal hernia and gastroesophageal reflux disease (GERD) requiring multiple endoscopies with expansion of the lower oesophageal sphincter for hypertension and a Toupet fundoplication. For the SSM group, seven minor malformations were diagnosed: one case each of phimosis, umbilical hernia and congenital torticollis, and two cases each of varus feet and lack of coverage of the hip.

In early childhood, one major malformation was found in the Global and SSM groups. For both groups, VSD was diagnosed in a child at a few months of life. In the Global group, the VSD was closed at the age of 2 years, whereas in the SSM group, the IVC persists but is not severe enough to warrant closure.
