# Supplementary material for: Does Embryo Culture Medium Influence the Health and Development of Children Born after In Vitro Fertilization?
Source: PLoS One. 2016 Mar 23;11(3):e0150857. doi: 10.1371/journal.pone.0150857 (PMC4805279; doi:10.1371/journal.pone.0150857)
Supplement: S3 File — (DOCX) [file pone.0150857.s004.docx]

**S3 File. Details on growth and medical concerns**

There was no difference between the groups for day-care hospitalization (25.0% of singletons of the Global group and 16.7% of singletons of the SSM group, *p* = 0.55). Almost half of the singletons from the SSM group required night-care hospitalization (43.3% but the difference with the children of the Global group (27.8%) was not significant (*p* = 0.21). Some children needed psychotherapy for a benign disease (16.7% in the Global group and 13.3% in the SSM group, *p* = 0.75). During their lives, nearly 50% of children in both groups had taken a treatment for a period exceeding one month (excluding homeopathy, vitamin D or fluorine). No difference was observed for chronic diseases (22.2% in the Global group and 20.0% in the SSM group, *p* = 1.0). The three cases of heart disease (2 in the Global group and 1 in the SSM group, *p* = 1.0) were non-serious VSD and no longer required specialist care at the age of 4 years. Regarding pulmonary diseases (11.1% of singletons of the Global group and 13.3% of singletons of the SSM group, *p* = 1.0), there was one case of severe asthma in the SSM group. The rest of the children suffered from mild asthma or an asthmatic equivalent (chronic cough). Regarding neurological diseases, one singleton from the Global group suffered from convulsive encephalopathy due to perinatal asphyxia caused by a tight nuchal cord: an anticonvulsant treatment was necessary up to the age of 5 months and specialized monitoring until the age of 3 years. At 4 years old, this child still suffered from minor motor sequelae (difficulty of coordination for climbing) but no trouble in learning. In the SSM group, one child was followed until the age of 18 months for macrocrania with hydrocephalus but without intracranial hypertension, and with normal neurological development. In both groups, serous otitis was responsible for two cases of transient deafness: one was treated bilaterally with ventilating tubes, the other is being treated medically. In the Global group, severe GERD complicated by behavioural anorexia was treated with Toupet fundoplication. Thanks to the operation, the child no longer suffers from GERD but should still be monitored for weight gain. Autoimmune neutropenia was also diagnosed in a singleton from the Global group at the age of 8 months. Continuous antibiotic treatment was required. The child was cured at the age of 2 years with no complications related to his neutropenia. In the SSM group, one child needed continuous antihistamine treatment for major eczema flare-ups. Moreover, there was no difference between the groups concerning surgical procedures (22.2% of singletons of the Global group and 16.7% of singletons of the SSM group, *p* = 0.76).
