# Supplementary material for: Does Embryo Culture Medium Influence the Health and Development of Children Born after In Vitro Fertilization?
Source: PLoS One. 2016 Mar 23;11(3):e0150857. doi: 10.1371/journal.pone.0150857 (PMC4805279; doi:10.1371/journal.pone.0150857)
Supplement: S1 Table — (DOCX) [file pone.0150857.s005.docx]

**S1 Table. Parental and cycle characteristics of singletons**

|  | *Global group (No. 42)* | *SSM group (No. 31)* | *p* |
| --- | --- | --- | --- |
| Maternal characteristics |  |  |  |
| Age (years) | 31.2 (4.0) | 30.5 (4.9) | 0.53 |
| Age ≥ 38 | 4 (9.5%) | 4 (12.9%) | 0.72 |
| Body Mass Index (BMI) | 22.0 (3.4) | 22.7 (4.4) | 0.46 |
| Smoking before pregnancy | 8 (19.1%) | 3 (10.0) | 0.34 |
| Smoking while pregnant | 3 (7.3%) | 1 (3.6%) | 0.64 |
| Number of pregnancy |  |  |  |
| 0 | 23 (54.8%) | 19 (61.3%) | 0.64 |
| ≥1 | 19 (45.2%) | 12 (38.7%) |  |
| Number of children |  |  |  |
| 0 | 26 (65.0%) | 22 (73.3%) | 0.53 |
| 1 | 10 (25.0%) | 7 (23.3%) |  |
| ≥2 | 4 (10.0%) | 1 (3.3%) |  |
| Paternal characteristics |  |  |  |
| Age (years) | 35.1 (4.6) | 35.0 (6.2) | 0.98 |
| Body Mass Index (BMI) | 24.7 (3.7) | 23.8 (3.2) | 0.28 |
| Current smoker | 18 (42.9%) | 19 (63.3) | 0.10 |
| Primary indication for IVF/ICSI treatment |  |  |  |
| Female factor | 14 (33.3%) | 6 (19.4%) | 0.25 |
| Male factor (OAT) | 15 (35.7%) | 17 (54.8%) |  |
| Mixed (female and male factors) | 8 (19.0%) | 7 (22.6%) |  |
| Idiopathic | 5 (11.9%) | 1 (3.2%) |  |
| Female factor |  |  |  |
| Ovulatory factor | 11 (50.0%) | 8 (61.5%) | 0.80 |
| Tubal factor | 8 (36.4%) | 3 (23.1%) |  |
| Endometriosis | 3 (13.6%) | 2 (15.4%) |  |
| Attempt number |  |  |  |
| 1 | 25 (59.5%) | 20 (64.5%) | 0.40 |
| 2 | 10 (23.8%) | 8 (25.8%) |  |
| 3 | 7 (16.7%) | 2 (6.5%) |  |
| 4 | 0 (0%) | 1 (1.4%) |  |

|  | *Global group (No. 42)* | *SSM group (No. 31)* | *p* |
| --- | --- | --- | --- |
| Technique |  |  |  |
| IVF | 17 (40.5%) | 10 (32.3%) | 0.47 |
| ICSI | 25 (59.5%) | 21 (67.7%) |  |
| Day of transfer |  |  |  |
| Transfer on Day 2 | 37 (88.1%) | 28 (90.3%) | 0.76 |
| Transfer on Day 3 | 5 (11.9%) | 3 (9.7%) |  |
| Single embryo transfer (SET) | 16 (38.1%) | 19 (61.3%) | 0.06 |

Data are presented as numbers (%) or mean (SD, standard deviation).

No.: number of cycles
