# Supplementary material for: Does Embryo Culture Medium Influence the Health and Development of Children Born after In Vitro Fertilization?
Source: PLoS One. 2016 Mar 23;11(3):e0150857. doi: 10.1371/journal.pone.0150857 (PMC4805279; doi:10.1371/journal.pone.0150857)
Supplement: S2 Table — (DOCX) [file pone.0150857.s006.docx]

**S2 Table. Maternal health during pregnancy**

|  | *Global group*  *(No. 39)* | *SSM group*  *(No. 31)* | *p* |
| --- | --- | --- | --- |
| Maternal chronic disease | 5 (12.8%) | 2 (6.7%) | 0.69 |
| Complications during pregnancy |  |  |  |
| Pregnancy-induced hypertension / pre-eclampsia | 3 (7.7%) | 2 (6.5%) | 0.81 |
| Gestational diabetes | 5 (12.8%) | 1 (3.2%) | 0.22 |
| Preterm labour | 6 (15.4%) | 4 (12.9%) | 1.00 |
| Placenta praevia | 3 (7.7%) | 1 (3.2%) | 0.62 |
| Induced labour | 7 (18.0%) | 7 (22.6%) | 0.77 |
| Delivery |  |  |  |
| Vaginal delivery (instrumental delivery included) | 30 (76.9%) | 24 (77.4%) | 0.36 |
| Planned Caesarean section | 1 (2.6%) | 3 (9.7%) |  |
| Emergency Caesarean section before labour | 3 (7.7%) | 3 (9.7%) |  |
| Emergency Caesarean section after labour | 5 (12.8%) | 1 (3.2%) |  |

Available data are presented as numbers (%).

No.: number of singletons
