# Supplementary material for: Does Embryo Culture Medium Influence the Health and Development of Children Born after In Vitro Fertilization?
Source: PLoS One. 2016 Mar 23;11(3):e0150857. doi: 10.1371/journal.pone.0150857 (PMC4805279; doi:10.1371/journal.pone.0150857)
Supplement: S3 Table — (DOCX) [file pone.0150857.s007.docx]

S3 Table. Neonatal data of singletons

|  | *Global group*  *(No. 40)** | *SSM Group*  *(No. 31)** |
| --- | --- | --- |
| Boys | 16 (40.0%) | 16 (51.6%) |
| Gestational age (GA) at birth | 38.5 (1.7) | 39.0 (1.5) |
| Pre-term birth (<37 weeks) | 4 (10.0%) | 1 (3.2%) |
| Hospitalization ≥ 7 days | 5 (13.2%) | 4 (12.9%) |
| Admission to neonatal ICU | 5 (12.8%) | 2 (6.5%) |
| Birthweight (g) | 3131 (505) | 3145 (448) |
| Low birthweight (<2500 g) | 4 (10.3%) | 2 (6.5%) |
| Low birthweight with GA ≥37 weeks | 2 (5.1%) | 1 (3.2%) |
| Small for GA (<10th perc.) | 3 (7.7%) | 2 (6.5%) |
| Very small for GA (<3rd perc.) | 1 (2.6%) | 1 (3.2%) |
| Large for GA (>90th perc.) | 2 (5.1%) | 2 (6.5%) |
| Very large for GA (>97th perc.) | 1 (2.6%) | 0 (0%) |

Available data are presented as numbers (%) or mean (SD, standard deviation).

No.: number of singletons

ICU: Intensive Care Unit

* Statistical analysis showed no difference between the groups, p>0.05
