# Supplementary material for: Does Embryo Culture Medium Influence the Health and Development of Children Born after In Vitro Fertilization?
Source: PLoS One. 2016 Mar 23;11(3):e0150857. doi: 10.1371/journal.pone.0150857 (PMC4805279; doi:10.1371/journal.pone.0150857)
Supplement: S4 Table — (DOCX) [file pone.0150857.s008.docx]

**S4 Table. Correlations between gender, term birth, mother and father socio-economic status with the CDI domains**

| Developmental age | Gender | Term Birth | Mother socio-economic status | Father socio-economic status |
| --- | --- | --- | --- | --- |
| Social | - 0.14 | 0.01 | -0.19 | - 0.15 |
| Self-help | - 0.14 | - 0.02 | - 0.03 | - 0.02 |
| Gross motor | - 0.08 | - 0.06 | - 0.07 | - 0.10 |
| Fine motor | - 0.04 | 0.13 | - 0.18 | - 0.02 |
| Expressive language | - 0.18 | 0.20 | - 0.31* | - 0.05 |
| Language comprehension | - 0.13 | 0.24 | - 0.35 * | - 0.14 |
| Letter knowledge | 0.04 | 0.20 | 0.00 | 0.01 |
| Number knowledge | - 0.11 | 0.22 | - 0.21 | 0.04 |

* Significant correlation (*p* < 0.05)
